# Rationality of Antimicrobial Prescriptions in Community Pharmacy Users

**DOI:** 10.1371/journal.pone.0141615

**Published:** 2015-10-30

**Authors:** Sara I. V. C. Lima, Rodrigo S. Diniz, Eryvaldo S. T. Egito, Paulo R. M. Azevedo, Antonio G. Oliveira, Ivonete B. Araujo

**Affiliations:** 1 Programa de Pós Graduação em Ciências da Saúde, Centro de Ciências da Saúde, Universidade Federal do Rio Grande do Norte, Natal-RN, Brazil; 2 Departamento de Farmácia, Centro de Educação e Saúde, Universidade Federal de Campina Grande, Cuité-PB, Brazil; 3 Departamento de Farmácia, Centro de Ciências da Saúde, Universidade Federal do Rio Grande do Norte, Natal-RN, Brazil; 4 Departamento de Estatística, Centro de Ciências Exatas e da Terra, Universidade Federal do Rio Grande do Norte, Natal-RN, Brazil; California Northstate University College of Medicine, UNITED STATES

## Abstract

**Background:**

Although there is a conflict between the treatment benefits for a single individual and society, restrictions on antibiotic use are needed to reduce the prevalence of resistance to these drugs, which is the main result of irrational use. Brazil, cataloged as a pharmemerging market, has implemented restrictive measures for the consumption of antibiotics. The objective of this study was to investigate the quality of antimicrobial prescriptions and user knowledge of their treatment with these drugs.

**Methods and Findings:**

A two-stage cross-sectional, combined and stratified survey of pharmacy users holding an antimicrobial prescription was conducted in the community between May and November 2014. A pharmacist analyzed each prescription for legibility and completeness, and applied a structured questionnaire to the users or their caregivers on their knowledge regarding treatment and user sociodemographic data. An estimated 29.3% of prescriptions had one or more illegible items, 91.3% had one or more missing items, and 29.0% had both illegible and missing items. Dosing schedule and patient identification were the most commonly unreadable items in prescriptions, 18.81% and 12.14%, respectively. The lack of complete patient identification occurred in 90.53% of the prescriptions. It is estimated that 40.3% of users have used antimicrobials without prescription and that 46.49% did not receive any guidance on the administration of the drug.

**Conclusions:**

Despite the measures taken by health authorities to restrict the misuse of antimicrobials, it was observed that prescribers still do not follow the criteria of current legislation, particularly relating to items needed for completion of the prescription. Moreover, users receive little information about their antimicrobial treatment.

## Introduction

The use of antimicrobials and the progressive emergence of resistant microorganisms have been discussed since the introduction of penicillin. Bacterial resistance has a negative impact on treatment results, increasing the death rate and hospitalization time around two-fold [1]. Thus, the improper use of these drugs culminates in drug-resistant infections and a rise in health costs that could be avoided [2].

According to the World Health Organization (WHO), the selective pressure that produces drug-resistant bacteria is also induced by the inappropriate use of antimicrobials [3]. Therefore, although there is a conflict between the treatment benefits for a single individual and for society, restrictions on the use of antimicrobials are necessary to reduce the resistance to these drugs [4], the main consequence of irrational use.

Brazil was catalogued by Intercontinental Marketing Services Health (IMS Health) as an pharmemerging country that, along with 20 others on the list, will account for two-thirds of global pharmaceutical growth between 2012 and 2017 [5]. Antimicrobials were one of the most widely consumed drugs in the country [6] and in November 2010 the National Agency of Sanitary Surveillance (ANVISA) regulated the control and sale of these drugs, requiring prescriptions to purchase this class of drugs, which had hitherto been sold freely, also establishing the mandatory prescription data, among other regulations involving products containing antimicrobials in its composition [7]. In 2011 the resolution was updated [8], but only in 2013 antimicrobials were included in the National System for Managing Controlled Products (SNGPC) to increase supervision on consumption [9]. With these regulations, it was expected that all pharmacies dispense antibiotics only if the prescription was presented. However, it is still possible to find pharmacies that circumvent the law.

Despite these measures, there was no information campaign regarding the risks of inappropriate use of these drugs for both prescribers and the general population. This fact was likely responsible for the slight reduction in antimicrobial consumption in the country, when compared to findings in Chile, where an education program was implemented [10]. A study conducted in northern Israel found a decline in the number of prescriptions as a result of multidisciplinary intervention groups, demonstrating that it was a good strategy for increasing the rational use of these drugs [11].

A number of studies have shown that the most common errors in the use of antimicrobials primarily involve treatment duration, prescription omissions or patient noncompliance, in addition to selecting antimicrobials without undergoing specific tests to aid diagnosis [12–15].

A number of factors that can significantly increase medication errors, causing harm to patients, are illegible and incomplete prescriptions [16]. Albarrak et al., in Saudi Arabia found that 23.63% of primary care prescriptions were incomplete and 21.6% illegible or difficult to read [17]. Calligaris et al. reported 23.9% illegibility and 29.9% incomplete prescriptions in an Italian hospital [18]. In a Brazilian study, 36% of prescriptions were considered illegible and 19.3% contained no information on dosage [19]. Another study, at a Brazilian hospital found 19.3% with partial or total illegibility in prescriptions of potentially dangerous medication [20].

Given the global concern over bacterial resistance, the need to promote the rational use of this class of drugs and the scarce number of studies on Brazilian community pharmacies, the aim of the present study was to analyze, based on information collected from users of community pharmacies, the quality of antimicrobial prescriptions in terms of legibility and completeness, as well as to investigate the knowledge of users regarding their treatment with antimicrobials.

## Methodology

The study was approved by the Research Ethics Committee of Universidade Federal do Rio Grande do Norte (CAAE: 17046213.1.0000.5292), in compliance with guidelines governing research with human beings, respecting the anonymity of participants and confidentiality of the information obtained.

This descriptive cross-sectional study was conducted in private community pharmacies (CP), with users of antimicrobials, covering the four districts of the city of Natal, the capital of the State of Rio Grande do Norte, in Northeastern Brazil.

Inclusion criteria were patients or caregivers acquiring antimicrobial medication at pharmacies between May and November 2014, aged 18 years or older, residents of Natal and who gave their informed consent. Patients or caregivers who were unable to respond to the questionnaire (S1 File) were excluded.

The sampling design was a combined stratified/two-stage random sample. In order to achieve representativity of all urban areas, 4 strata were defined, matching the 4 geographical districts into which the city is divided. In each stratum, a sample of community pharmacies were randomly selected in proportion to the total number of registered establishments in the corresponding district. Of the 18 pharmacies selected, 5 belonged to the eastern, 4 to the northern, 2 to the western and 7 to the southern district. Thus, the first stage consisted of a simple random sample of pharmacies in each stratum. Community pharmacies whose pharmacist was absent on two consecutive visits and those that did not sell antimicrobials were excluded. The second stage consisted of a consecutive sample of users of those pharmacies selected in the first stage. To standardize the data collected, the time spent in each community pharmacy was established as 5 working days, in the morning and afternoon.

All the interviews were conducted by the same researcher. Even though pharmaceutical counseling is not a common practice in Brazil, the questionnaire was always applied before any contact between users or caregivers and the community pharmacist. A structured questionnaire used in the interviews was divided into 3 blocks: (i) the first block recorded an evaluation by the researcher in charge of five items in the prescription, regarding legibility and completeness; (ii) the second block had nine closed questions about the knowledge of patients or caregivers regarding the treatment; finally (iii) the third block, containing 4 questions, collected sociodemographic data.

The questionnaire underwent prior pilot testing to assess its suitability in obtaining the desired information. Given that the changes introduced in the questionnaire were of little relevance, participants in pilot study, who have also gave written informed consent to participate, were included in the final sample.

Analysis of prescriptions was conducted solely by the researcher in charge. Items were assessed as illegible or incomplete based on a checklist prepared according to current legislation [8]. An item was classified as illegible if the researcher in charge was not able to understand its meaning after a first reading of the prescription. Each of the eight mandatory items determined by the law were assessed as present or absent, and legible or not.

In calculating prevalence estimates, sample weights wereused. A sample weight is the inverse of the probability that each subject of the sample is selected to the study. These were calculated based on the population census data of 2010 [21], and on the stratification and the two stage design. The primary sampling units were the pharmacies. For the first stage, sampling without replacement was used and correction for a finite population was based on the number of pharmacies in each district/stratum.

Sample size was calculated as 384 subjects, a number that ensures a maximum error of the estimates of 5% with a 95% confidence level for simple random sampling. This result was obtained by solving the expression z_0.95_^2^ × π(1 – π) / ε^2^ (= 1.96^2^ × 0.5 × 0.5 / 0.05^2^). Confidence intervals were calculated with the Jacknife method, a general method of estimation commonly used in complex survey designs.

The statistical program used was STATA 12.0 (Stata Corporation, College Station, TX). The results are presented as point estimates and 95% confidence intervals (95% CI).

## Results

Data collection was conducted between May and November 2014. A total of 340 individuals were invited to take part prospectively in the study, 323 of whom responded to the questionnaire, resulting in 5% sample loss.

The sociodemographic characteristics of the sample are depicted in Table 1. A higher proportion of the sample (69.66%) was adults, and 57.86% were females. A majority lived with other individuals and had education at high school level or greater.

**Table 1:** Sociodemographic characteristics of the study sample.

	n	%
Users	323	100.0
Patients	267	82,66
Caregivers	56	17,34
Female Sex	186	57.86
Users		
Children and adolescents (up to 19 years)	38	11,76
Adults	246	76,16
Elderly (older than 65 years)	39	12.08
Residing with others	301	93.16
Education: high school or greater	200	61.91

Amoxicillin was the most prescribed antibiotic (15.8%), followed by azithromycin (11.46%) and ciprofloxacin (10.48%); omission of the prescriber’s specialty was observed on 38.08% of prescriptions. Most prescriptions were of private origin, and only 33.8% came from the public health system (SUS).

Table 2 contains data on prescription legibility and completeness. The dosing schedule and patient identification were the most commonly unreadable items in prescriptions, with a frequency of 18.81% and 12.14%, respectively. Complete identification of patients was lacking in 90.53% of prescriptions, followed by the name of the medication, according to the Common Brazilian Denomination (CBD), which was absent in 46.55%. A total of 28.95% of users had illegible (at least 1 illegible item) and incomplete (at least 1 item missing) prescriptions.

**Table 2:** Analysis of antimicrobial prescriptions.

Variable	%*	95% CI*
**Illegibility**		
Prescription copy	4.74	1.25–8.24
Patient identification	12.14	6.41–17.87
Name of the medication	4.44	2.23–6.66
Dosage of the medication	4.12	1.18–7.05
Dosing schedule	18.81	7.75–29.88
Amount of medication	2.67	0–5.32
Identification of prescriber	2.92	1.10–4.74
Date of issue	0.88	0–1.8
Illegible prescriptions	29.28	17.80–40.75
**Incompleteness**		
Prescription copy	7.46	2.15–12.77
Patient identification	90.53	80.02–100
Name of the medication (CDB)	46.55	29.42–63.67
Dosage of medication	23.11	14.10–32.12
Dosing schedule	2.19	0.87–5.27
Amount of medication	19.59	15.12–24.05
Identification of prescriber	2.30	0.15–4.75
Date of Issue	7.20	2–12.41
Incomplete prescriptions	91.25	81.14–100
Illegible and incomplete prescriptions	28.95	17.52–40.37

* Percent estimates and CI 95% obtained by weighting.

Table 3 presents data on the knowledge of users or their caregivers regarding treatment with antimicrobials, before any contact with the pharmacist, as mentioned. It was found that 40.3% of users had already used antimicrobials with no medical prescription and 46.49% received no orientation on drug administration. More than half of those interviewed (51.51%) stored their medication improperly in the kitchen.

**Table 3:** Knowledge of users regarding antimicrobial treatment.

Variable	%*	95% CI*
Repeated use of the medication for the same disease	35.60	27.31–43.89
No knowledge duration of treatment	23.12	18.87–27.37
Occurrence of ADR** with antibiotics	10.32	5.63–15.02
Use of medication without prescription	40.3	34.37–46.23
When forgets to take medication, does not do so upon remembering	24.7	18.75–30.64
Did not receive information on administration	46.49	43.55–49.43
Stores medication in the kitchen	51.51	45.91–57.11

* Percent estimates and CI 95% obtained by weighting.

** ADR: Adverse Drug Reaction

With respect to treatment, 20.18% of those interviewed reported that they did not always follow the recommended treatment duration and 21.13% did not take their medication according to the administration schedule. In relation to general medication precautions, 34.22% of users did not always check the drug expiration date; however, 95.01% reported never having taken it after this date. A total of 36.08% did not always check the prescription before taking their medication. Most of the users or caregivers (74.4%) claimed they always kept medications in their original packaging and 94.19% stored them out of reach of children and animals.

## Discussion

In this study, we present the results of a survey on the quality of antimicrobial prescriptions and on user knowledge of their treatment with these drugs based on information collected in questionnaire-based interviews of a probability sample of 323 subjects which were conducted at community pharmacies. Thus, the results can be taken as representative of the population that uses community pharmacies in the different areas of Natal, Brazil, a state capital with over 800,000 inhabitants.

Most studies published on prescription quality are conducted in hospital settings, where it is easier to obtain patient data. Our study ensured good population coverage by sampling18 CPs in every district of the city, an improvement over those studies performed in a singlehospital [17, 18, 20] or CP [19]. Furthermore, patient interviews were conducted prospectively, whereas other studies have only analyzed prescriptions [17–20]. We focused our study on antimicrobial prescriptions, because inappropriate prescription of this class of drugs is a matter of public health concern, since incorrect individual use may compromise community health[4].

The RDC 20, of May 5, 2011 [8], was the last update of the regulation for antimicrobials sale, which were non-prescription drugs until the year 2010. The regulation establishes that for dispensing this drug class, the presentation of a prescription is essential or the sale cannot be performed. This law also introduced mandatory requests concerning the prescription itself. Indeed, according to the law, a prescription must contain 8 items: (i) copy of the prescription, (ii) the complete patient identification (name, age and sex), (iii) the medication name, according to the Brazilian Common Denomination (DCB), (iv) the dosage and (v) dosing schedule, (vi) the amount of drug to be dispensed, (vii) the identification of the prescribing physician and (viii) the prescription date. Also, the prescription will have a validity date of only 10 days. None of the items may be erased. If a recipe is incomplete, the pharmacist can ask the patient to return to the prescriber. However, if the missing items are easy to be collected by the pharmacist, the drug can be sold. Through SNGPC, the data of all the prescriptions are sent to Anvisa. The prescription must be stored for 2 years in the pharmacy. The health authority of each city is responsible for the enforcement of this law by evaluating the recipes kept in the pharmacies. Fines would be issued whenever prescriptions are found in violation of the regulations.

It is known that illegible handwriting, and omitted or incomplete information leads to numerous administration errors [22,23]. The results of this study have shown that 91.25% of prescriptions had at least one missing item and that 29.28% contained at least one illegible item. The most frequently illegible item was dosage (61 of 94 illegible prescriptions), essential information to guarantee the correct use of medication and therefore, the rational use of antimicrobials. The most common incomplete item was the complete patient information. According to the legislation, the doctor should insert full name, age and sex of the patient, but in almost all prescriptions only the name was present. Although it is possible to collect this information in the pharmacy, upon purchase, the patient himself is not always the buyer. In addition, for the WHO the information about age is of utmost importance, especially for children and elderly, since many drugs have usage restrictions for these ages [24].

Calligaris et al. on their study on hospital prescriptions suggested that global illegibility and incompleteness values of more than 20% are unacceptably high [18]. Our study shows that this target is far from being reached three years after legal requirements for antimicrobial prescription have been in force [8] and that further efforts should be used to foster, prescribers adherence to prescription guidelines and regulations in order to guarantee patient safety. The increasing use of electronic prescription systems should be an important step to decreasing the problems of illegibility and omissions in prescriptions [17,18].

With respect to the knowledge of users or their caregivers, nearly half of those about to initiate treatment did not receive any information regarding antimicrobial administration. For exemple, most oral antimicrobials are not affected by the concomitant ingestion of food, but ciprofloxacin, the third most prescribed medication used without orientation, should not be administered with dairy products, due to the formation of chelates and decreased bioavailability [25]. Another important information is the use of over-the-counter medication, such as antacids, which also lead to the formation of chelates when administered with quinolones.

A total of 23.12% of users are unaware of treatment duration, an important information for rational use of antimicrobials. Moreover, 20.18% did not always comply with recommended treatment duration. This demonstrates the users’ lack of knowledge regarding antimicrobials in terms of the importance of treatment duration, as has also been reported by Shahadeh et al [15], who found that one-third of users do not believe that the effectiveness of antimicrobials depends on treatment duration.

The user information presented here, as well as the fact that 35.6% reported having used the same antimicrobial for the same health problem and that 24.7% did not take their medication as soon as they remembered or at the appointed hour, is indicative of a communication problem between prescribersand users. Accordingly, healthcare professionals must devote time to explain clearly and completely to patients or caregivers the details of drug use, in order to minimize administration errors and achieve effective and safe treatment.

Although it has been reported that some physicians consider that not prescribing antibiotics might have a negative effect on the doctor-patient relationship [26], restrictions on the acquisition of antimicrobials have been implemented in order to eliminate, or at least decrease, self-medication. This is considered beneficial in reducing the prevalence of bacterial resistance [4]. However, in our population 40.3% of users have already used antimicrobials without a prescription, sometimes by purchasing them without one and some other times by using medication leftovers from previous treatments. In order to promote the rational use of these drugs, avoiding their use without medical supervision, educational campaigns aimed at the non-recycling or stocking of antimicrobials should be conducted.

Most of the study population (61.91%) had a good educational level, which should indicate adequate knowledge regarding the use and precautions required with medication. However, there was a contradiction between knowledge and behavior, similar to that detected by Shahadeh et al [15]. Although most users (74.4%) reported keeping their medication in its original packaging and out of reach of children and animals (94.19%), they also demonstrated negligence with drugs, primarily in terms of storage, since 51.51% kept their medication in the kitchen. Furthermore, 34.22% did not always check the expiry date before use and 36.08% did not verify whether the medication they were taking was the same as that stipulated on the prescription. These findings show the lack of importance given to medication precautions and proper storage, which increases the risk of it becoming inappropriate for administration even before the expiration date.

This work pointed out several important aspects. For example, our methodology contributes importantly to the validity of the results because it includes the probability sample calculation in the community pharmacies, based on a complex sampling plan. The methodology also covers important aspects such as prospective user interviews, direct assessment of pharmacist prescriptions and low rate of non-respondents. Additionally, although the target planned sample size of 384 subjects was not reached because of the time constraints on the conclusion of the researce, it does not compromise the validity of the estimates because only a modest impact on the precision of the study, traduced by an increase of 0,5% on the maximum error of the estimates was observed.

However, in spite of the important findings of this research, some limitations of the study can be observed. For example, the results concern only the city of Natal, Brazil. Therefore, generalization to other populations must be made carefully, as differences in demography, education, legislation, tradition and in prescription and dispensing practices may have impact on the results. Another limitation is the relatively small sample size, which is the minimum acceptable for a population survey, since a larger sample size would provide greater robustness to the results. The appropriateness of the prescription could not be assessed as we had no information regarding the indication for the antimicrobial medication. We could not verify what information was actually given to the patient, or to the caregiver, by the prescribing physician, and we could only evaluate the information that was perceived by the subject. Finally, we assume that the questions asked in the interview have face validity, but our questionnaire has had no formal validation and, as such, it is not certain that all subjects understood what was being asked and have provided reliable answers.

## Conclusion

Despite the measures adopted by sanitary surveillance authorities to restrict the abusive use of antimicrobials, prescribers still fail to comply with legislation, mainly in regard to the items required for a complete a prescription. Moreover, users receive little information on their treatment and precautions with medication. It is therefore suggested that educational campaigns and evidence-based guidelines be implemented to increase the quality of prescriptions and improve communication between health professionals and users regarding the correct use of medication.

## Supporting Information

S1 File Questionnaire used for data collection. (DOCX)
