# Supplementary material for: Rationality of Antimicrobial Prescriptions in Community Pharmacy Users
Source: PLoS One. 2015 Oct 30;10(10):e0141615. doi: 10.1371/journal.pone.0141615 (PMC4627736; doi:10.1371/journal.pone.0141615)
Supplement: S1 File — (DOCX) [file pone.0141615.s001.docx]

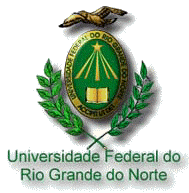


**MINISTÉRIO DA EDUCAÇÃO**

**UNIVERSIDADE FEDERAL DO RIO GRANDE DO NORTE**

**CENTRO DE CIÊNCIAS DA SAÚDE**

**PROGRAMA DE PÓS-GRADUAÇÃO EM CIÊNCIAS DA SAÚDE**

**Questionnaire for data collection**

01. Antibiotic´s name.

02. Specialty of the prescriber

_____________________________________________________________________

03. Origin of the prescription:

a) ( ) Private

b) ( ) Public system

04. Which of the following items are illegible?

a) ( ) Prescription copy

b) ( ) Patient identification

c) ( ) Name of the medication

d) ( ) Dosage of the medication

e) ( ) Dosing schedule

f) ( ) Amount of medication

g) ( ) Identification of prescriber

h) ( ) Date of issue

i) ( ) The prescription is legible

05. Which of the following items are incomplete?

a) ( ) Prescription copy

b) ( ) Patient identification

c) ( ) Name of the medication

d) ( ) Dosage of the medication

e) ( ) Dosing schedule

f) ( ) Amount of medication

g) ( ) Identification of prescriber

h) ( ) Date of issue

i) ( ) The prescription is legible

06. Have you already used this drug for the same health problem?

a) ( ) Yes

b) ( ) No

c) ( ) I don’t remember

07. Have you been told how many days you will take this medicine?

a) ( ) Yes. How many? ____________

b) ( ) No

c) ( ) I don’t remember

08. Do you usually follow the guidelines of your treatment?

a) Regarding the length of time

( ) Never ( ) Rarely ( ) Sometimes ( ) Often ( ) Always

b) Regarding the schedule taking the medicine

( ) Never ( ) Rarely ( ) Sometimes ( ) Often ( ) Always

c) Regarding the dosage to be taken in each time

( ) Never ( ) Rarely ( ) Sometimes ( ) Often ( ) Always

09. Antibiotics can cause adverse drug reactions like allergies, pain in the belly, earaches, among others. Have you ever had any of those?

1. ( ) Yes. Which ones? _________________________
2. ( ) No
3. ( ) I don’t know

10. Have you ever used any leftover antibiotics from prior treatments, without going back to the doctor?

a) ( ) Yes

b) ( ) No

c) ( ) I don’t remember

11. What do you usually do when you forget to take the medication at the right time?

a) ( ) I take it exactly when I remember

b) ( ) Wait for the next schedule

c) ( ) I never forget

12. According to the presented scale, how often do you take the following precautions before taking this medicine:

a) Always check the validity of the medicines before using

( ) Never ( ) Rarely ( ) Sometimes ( ) Often ( ) Always

b) Take any medication out of date

( ) Never ( ) Rarely ( ) Sometimes ( ) Often ( ) Always

c) Read the prescription before using

( ) Never ( ) Rarely ( ) Sometimes ( ) Often ( ) Always

d) Always keep the medicine in the original box after starting its use

( ) Never ( ) Rarely ( ) Sometimes ( ) Often ( ) Always

e) Keep the medications away from children and animals

( ) Never ( ) Rarely ( ) Sometimes ( ) Often ( ) Always

13. Have you been referred to take the medication by one of these ways?

a) ( ) With any liquid

b) ( ) Only with water

c) ( ) Before meals

d) ( ) With meals

e) ( ) None of the options above (injectable or topical medicine)

f) ( ) I didn’t receive any counseling

g) ( ) I don’t remember

14. Where do you usually store your medicines:

a) ( ) Bedroom

b) ( ) Kitchen

c) ( ) Bathroom

d) ( ) Living room

e) ( ) Anywhere

15. Gender

1. ( ) male
2. ( ) female

16. Age ______ years

17. Residing:

1. ( ) Alone
2. ( ) With others

18. Level of education:_________________
